# Identification of Risk Factors for Postoperative Hypotension Following Transurethral Bladder Tumor Resection Performed With Oral 5-Aminolevulinic Acid: A Multivariate Analysis of a Single-Center Retrospective Cohort Study

**DOI:** 10.7759/cureus.82112

**Published:** 2025-04-11

**Authors:** Takashi Oshikawa, Toyoaki Maruta, Go Otao, Isao Tsuneyoshi

**Affiliations:** 1 Department of Anesthesiology, University of Miyazaki Hospital, Miyazaki, JPN

**Keywords:** 5-aminolevulinic acid, bladder tumor, multivariate analysis, postoperative hypotension, spinal anesthesia, transurethral resection

## Abstract

Background

Transurethral resection of bladder tumors (TUR-Bt) using 5-aminolevulinic acid (5-ALA) is increasingly performed to visualize tumors. However, oral administration of 5-ALA frequently induces perioperative hypotension. Although several risk factors for intraoperative hypotension have been reported, those associated with postoperative hypotension have not yet been identified. We retrospectively evaluated risk factors for postoperative hypotension following TUR-Bt with 5-ALA administration.

Methods

The enrolled participants were patients who underwent TUR-Bt with 5-ALA under general or spinal anesthesia between July 2020 and December 2023. Patients who developed postoperative hypotension or used postoperative vasopressors were assigned to the hypotension group, and those who did not were assigned to the non-hypotension group. Postoperative mean blood pressure (mBP) was sampled from the electronic medical record at 1, 2, 3 and 6 hours after surgery. Postoperative hypotension was defined as an mBP of < 70 mmHg, noted at least once in the electronic medical records. Risk factors were identified using multivariate analysis. In addition, a subset of spinal anesthesia cases was similarly analyzed.

Results

Among 111 patients who underwent TUR-Bt with 5-ALA under general or spinal anesthesia, 46 and 65 were categorized into the hypotension and non-hypotension groups, respectively. Risk factors identified were estimated glomerular filtration rate (eGFR) ≤ 45-60 mL/min/1.73 m^2^, eGFR < 45 mL/min/1.73 m^2^, and mBP < 95 mmHg upon entering the operating room (odds ratio (OR) 3.026, 95% confidence interval (CI) 1.140-8.003, *P = *0.027; OR 4.851, 95% CI 1.550-15.177, *P* = 0.007; and OR 2.443, 95% CI 1.018-5.865, *P* = 0.046, respectively). From the 111 patients, 88 underwent spinal anesthesia (38 hypotensive, 50 non-hypotensive). Risk factors identified among these patients were body mass index, eGFR ≤ 45-60 mL/min/1.73 m^2^, eGFR < 45 mL/min/1.73 m^2^, and mBP < 95 mmHg upon entering the operating room (OR 1.290, 95% CI 1.079-1.542, *P* = 0.006; OR 3.757, 95% CI 1.153-12.249, *P* = 0.029; OR 7.295, 95% CI 01.804-29.501, *P* = 0.006; and OR 3.134, 95% CI 1.061-9.262, *P* = 0.039, respectively).

Conclusion

Regardless of anesthesia method, impaired renal function increased postoperative hypotension, whereas higher blood pressure before anesthesia was less likely to result in postoperative hypotension.

## Introduction

Transurethral resection of bladder tumors (TUR-Bt) using 5-aminolevulinic acid (5-ALA) is commonly performed to visualize tumors. However, perioperative hypotension occurs frequently during surgery when 5-ALA is administered [1-9]. Although several risk factors for intraoperative hypotension have been reported, those associated with postoperative hypotension have not yet been identified. These factors are also of clinical interest owing to their potential impact on patient outcomes. Postoperative hypotension is associated with myocardial infarction, myocardial injury after noncardiac surgery, and acute kidney injury, and may lead to death [10-12].

Consequently, in the present study, we retrospectively examined the dependent risk factors for postoperative hypotension in patients who underwent TUR-Bt with administration of oral 5-ALA under general or spinal anesthesia. With regard to the anesthetic technique, general anesthesia is an independent risk factor for intraoperative hypotension, compared with spinal anesthesia [13-18]; however, perioperative hypotension owing to oral 5-ALA has also been observed in patients receiving spinal anesthesia [19,20]. Furthermore, there are other factors unique to spinal anesthesia, such as the drug and method used. Therefore, we also examined the same risk factors for postoperative hypotension in a subset of patients who underwent spinal anesthesia.

## Materials and methods

Study population

This study was approved by the Ethics Committee of the University of Miyazaki Hospital (approval number O-1211). The need for informed consent from patients was waived owing to the retrospective nature of the study. The enrolled participants were patients who underwent TUR-Bt with 5-ALA for photodynamic diagnosis, between July 2020 and December 2023 at the University of Miyazaki Hospital. The exclusion criteria for patients were absence of records of vital signs before 5-ALA administration, additional surgery other than TUR-Bt, reoperation owing to postoperative bleeding, surgery lasting for more than 120 min, or absence of postoperative blood pressure records. First, independent risk factors for postoperative hypotension were analyzed in all patients undergoing TUR-Bt with 5-ALA under general or spinal anesthesia. Subsequently, risk factors were analyzed in the same manner in a subset of patients who received spinal anesthesia.

All patients received oral 5-ALA (20 mg/kg; Alaglio®, SBI Pharmaceuticals Co., Ltd., Tokyo, Japan) 2−4 hours before surgery. After the patient was admitted to the operating room, standard monitoring was performed, including electrocardiography, noninvasive blood pressure measurements, and pulse oximetry. Anesthesia details and hypotension treatments were determined by an individual anesthesiologist based on the condition of the patient.

Data collection

The following data were collected from electronic medical and anesthesia records: age; sex; height; weight; body mass index (BMI); American Society of Anesthesiologists physical status classification (ASA-PS); preoperative hemoglobin concentration and hematocrit; preoperative estimated glomerular filtration rate (eGFR; mL/min/1.73 m^2^); comorbidities (hypertension, heart disease, diabetes, end stage of renal disease); use of renin-angiotensin system inhibitors (RASI), calcium channel blockers, α- and β-blockers, diuretics; anesthetic technique (general anesthesia or spinal anesthesia); operation and anesthesia time; infusion volume (preoperative infusion volume, intraoperative infusion volume, postoperative infusion volume up to 6 h after surgery); use of ephedrine, phenylephrine, dopamine, dobutamine, and noradrenaline; mean blood pressure (mBP) before 5-ALA administration and on entry into the operating room, mBP from induction of anesthesia to the beginning of surgery, mBP after the beginning of surgery, mBP on leaving the operating room, and mBP at 1, 2, 3, and 6 h after surgery; postoperative use of vasopressors; postoperative complications; and in cases of spinal anesthesia, the lumbar intervertebral space used, specific baricity, and volume of the drug solution used, and preoperative and postoperative anesthesia levels. Cardiac disease was defined as atrial fibrillation, pacemaker implantation, moderate or severe valvular disease, prior percutaneous coronary intervention or coronary artery bypass surgery, or prior heart failure.

Postoperative mBP was obtained from electronic medical records 1, 2, 3, and 6 h after intervention. Based on a previous report that investigated the association between postoperative hypotension and myocardial damage [12], postoperative hypotension was defined as an mBP of < 70 mmHg, noted at least once in the electronic medical records. Patients who developed postoperative hypotension or used postoperative vasopressors were assigned to the hypotension group. All patients were divided into two groups: the non-hypotension and hypotension groups.

Statistical analysis

Data are expressed as the median (interquartile range). mBP trends are expressed as the mean ± standard deviation (SD). For between-group comparisons, Fisher’s exact probability test was used for categorical variables, and the Mann-Whitney U test was used for continuous variables. Univariate and multivariate analyses were performed using mixed models; that is, patients who experienced two or more surgeries during the study period were also included. Multivariate analysis using the forced entry method was performed for factors that were significantly different on univariate analysis. Intraoperative blood pressure was not included in the univariate and multivariate analyses because the study aimed to predict the occurrence of postoperative hypotension from information other than intraoperative blood pressure. Intraoperative fluid volume and intraoperative vasopressors were not included in the multivariate analysis because they were expected to reflect intraoperative hypotension. Statistical significance was set at *P* < 0.05. Statistical analyses were performed using SPSS Statistics for Windows version 29.0 (IBM Corp., Armonk, NY, USA).

## Results

**Figure 1:**
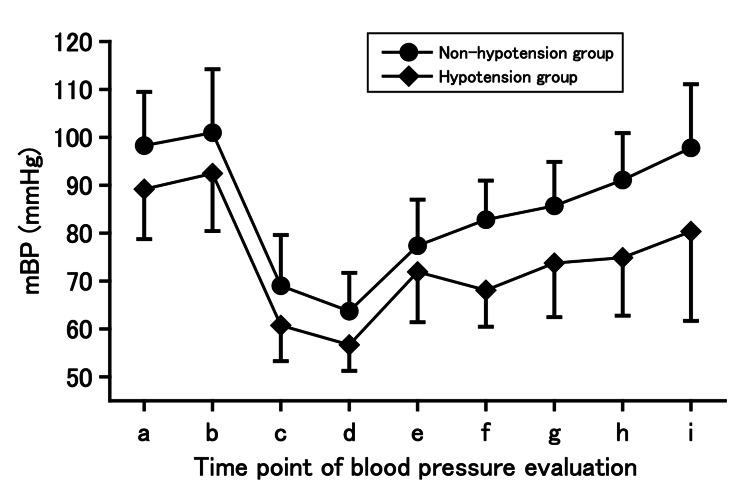
Trends of mean blood pressure in the non-hypotension and hypotension groups of all patients who underwent general or spinal anesthesia. The non-hypotension group: n = 65, and hypotension group: n = 46. In this study using mixed models, patients who underwent two or more TUR-Bt during the study period were also included. For duplicate cases, data from the first case was used. Data are expressed as mean ± standard deviation. mBP, mean blood pressure. Timepoints: a, before administration of 5-aminolevulinic acid; b, upon entering the operating room; c, minimum value before surgery; d, minimum value after starting surgery; e, upon leaving the operating room; f, 1 h after surgery; g, 2 h after surgery; h, 3 h after surgery; and i, 6 h after surgery.

In this study, a mixed model was used for univariate and multivariate analysis. Patients who underwent two or more TUR-Bt during the study period were also included, so the number of patients and cases differed. A total of 126 patients who underwent TUR-Bt with 5-ALA treatment were enrolled in the initial study: 27 patients received general anesthesia and 99 patients received spinal anesthesia. Among the patients who underwent general anesthesia, four were excluded and 23 were included in the study (28 cases). Among the 99 patients who underwent spinal anesthesia, 11 were excluded and 88 included (105 cases). Thus, a total of 111 patients were included (133 cases in total). Patients with a postoperative mBP of < 70 mmHg and those who used postoperative vasopressors were defined as the hypotension group. Thus, 65 and 46 patients comprised the non-hypotension and hypotension groups, respectively. Table 1 shows the patient characteristics and perioperative information of the two groups. In the between-group comparisons, significant differences were found in eGFR, intraoperative infusion volume, phenylephrine dose, mBP before 5-ALA administration, mBP upon entry into the operating room (before anesthesia induction), minimum mBP before surgery (after anesthesia induction), minimum mBP after starting surgery, mBP upon leaving the operating room, mBP at different time points, 1, 2, 3, and 6 h, after surgery, and use of postoperative vasopressors. The perioperative mBP trends in each group are shown in Figure 1.

**Table 1:** Patient characteristics and perioperative data In this study using mixed models, patients who underwent two or more TUR-Bt during the study period were also included. For duplicate cases, data from the first case was used. Fisher’s exact probability test was used for categorical variables, and the Mann–Whitney U test was used for continuous variables. 5-ALA, 5-aminolevulinic acid; ASA-PS, American Society of Anesthesiologist physical status classification; BMI, body mass index; eGFR, estimated glomerular filtration rate; IQR, interquartile range; mBP, mean blood pressure; *n*, number; RASI, renin–angiotensin system inhibitor.

Variables	Non-hypotension group		Hypotension group		*P*-value
	n	Data	n	Data	
Patient characteristics					
Age (years), median (IQR)	65	73.0 (66.5–81.0)	46	73.0 (68.0–80.0)	0.579
Sex	65		46		0.296
Male, n (%)		52 (80.0)		41 (89.1)	
Female, n (%)		13 (20.0)		5 (10.9)	
Height (cm), median (IQR)	65	163.4 (157.0–171.7)	46	163.8 (159.6–167.6)	0.790
Weight (kg), median (IQR)	65	61.0 (52.4–72.5)	46	65.5 (57.3–68.2)	0.088
BMI (kg/m^2^), median (IQR)	65	23.4 (20.6–25.8)	46	24.1 (22.4–25.6)	0.197
ASA-PS	65		46		0.618
1, n (%)		1 (1.5)		2 (4.3)	
2, n (%)		58 (89.2)		41 (89.1)	
3, n (%)		6 (9.2)		3 (6.5)	
Hemoglobin (g/dL), median (IQR)	65	14.1 (12.6–15.5)	46	13.7 (12.3–15.2)	0.320
Hematocrit (%), median (IQR)	65	42.1 (39.3–45.6)	46	41.7 (37.4–44.9)	0.417
eGFR (mL/min/1.73 m^2^), median (IQR)	64	64.5 (54.0–74.8)	44	54.0 (43.0–68.5)	0.008
≥ 60, n (%)		40 (62.5)		16 (36.4)	0.017
45–60, n (%)		15 (23.4)		14 (31.8)	
< 45, n (%)		9 (14.1)		14 (31.8)	
Comorbidity	65		46		
Hypertension, n (%)		39 (60.0)		26 (56.5)	0.845
Heart disease, n (%)		7 (10.8)		5 (10.9)	> 0.999
Diabetes mellitus, n (%)		12 (18.5)		8 (17.4)	> 0.999
End stage of renal disease, n (%)		1 (1.5)		2 (4.3)	0.569
Medication	65		46		
RASI, n (%)		27 (41.5)		17 (37.0)	0.696
Calcium channel blocker, n (%)		32 (49.2)		23 (50.0)	> 0.999
α-blocker, n (%)		6 (9.2)		5 (10.9)	0.760
β-blocker, n (%)		7 (10.8)		4 (8.7)	> 0.999
Diuretic, n (%)		5 (7.7)		2 (4.3)	0.697
Perioperative data					
Anesthesia method	65		46		0.640
General, n (%)		15 (23.1)		8 (17.4)	
Spinal, n (%)		50 (76.9)		38 (82.6)	
Operation time (min), median (IQR)	65	50.0 (35.0–64.3)	46	48.0 (28.8–69.0)	0.635
Anesthesia time (min), median (IQR)	65	77.0 (60.0–93.5)	46	77.5 (50.8–95.0)	0.722
Infusion volume	65		46		
Preoperative infusion volume (mL), median (IQR)		0.0 (0.0–0.0)		0.0 (0.0–162.5)	0.072
Intraoperative infusion volume (mL), median (IQR)		700.0 (500.0–900.0)		825.0 (637.5–1200.0)	0.024
Postoperative infusion volume up to 6 h (mL), median (IQR)		480.0 (480.0–480.0)		480.0 (480.0–480.0)	0.806
Intraoperative vasopressor	65		46		
Ephedrine (mg), median (IQR)		8.0 (0.0–12.0)		12.0 (0.0–16.0)	0.204
Phenylephrine (mg), median (IQR)		0.0 (0.0–0.1)		0.2 (0.0–0.6)	0.006
Dopamine, n (%)		0.0 (0.0)		3.0 (7.3)	0.080
Dobutamine, n (%)		1.0 (1.9)		2 (5.0)	0.573
Norepinephrine, n (%)		3.0 (5.5)		6.0 (14.3)	0.163
Analgesic drug	65		46		
Fentanyl, n (%)		16.0 (24.6)		9.0 (19.6)	0.646
Pentazocine, n (%)		13.0 (26.0)		9 (23.7)	> 0.999
Blood pressure	65		46		
mBP before administration of 5-ALA, median (IQR)		96.3 (91.5–104.3)		91.8 (79.4–95.8)	< 0.001
mBP upon entering the operating room, median (IQR)		101.0 (90.8–107.5)		92.5 (85.0–101.2)	0.001
Minimum mBP before starting operation, median (IQR)		65.3 (58.0–74.7)		60.7 (56.0–65.4)	0.003
Minimum mBP after starting operation, median (IQR)		62.3 (58.2–66.6)		56.5 (52.8–59.8)	< 0.001
mBP upon leaving the operating room, median (IQR)		78.3 (71.7–88.5)		70.2 (64.4–75.8)	< 0.001
mBP 1 h after surgery, median (IQR)		82.3 (76.2–89.2)		67.8 (62.6–73.3)	< 0.001
mBP 2 h after surgery, median (IQR)		85.0 (78.2–91.3)		73.5 (68.5–81.1)	< 0.001
mBP 3 h after surgery, median (IQR)		87.7 (82.0–97.3)		71.0 (66.2–82.4)	< 0.001
mBP 6 h after surgery, median (IQR)		95.0 (85.3–104.2)		80.7 (72.0–91.7)	< 0.001
Use of postoperative vasopressors, n (%)	65	0.0 (0.0)	46	13.0 (28.3)	< 0.001
Postoperative complications, n (%)	65	6.0 (9.2)	46	7.0 (15.2)	0.379
Classification		Nausea and vomiting, 7 cases; delirium, 1 case		Nausea and vomiting, 4 cases; delirium, 1 case; headache, 2 cases	

The results of the univariate and multivariate analyses are shown in Tables 2-3. Univariate analysis revealed significant differences in eGFR, intraoperative fluid volume, mBP before 5-ALA administration, and mBP upon entry into the operating room. Multivariate analysis was performed on these factors except for intraoperative fluid volume, and results revealed that eGFR ≤ 45-60 mL/min/1.73 m^2^, eGFR < 45 mL/min/1.73 m^2^, and mBP < 95 mmHg upon entry into the operating room were significant risk factors for postoperative hypotension.

**Table 2:** Univariate analysis of predictive factors of postoperative hypotension Univariate analyses were performed using mixed models. 5-ALA, 5-aminolevulinic acid; ASA-PS, American Society of Anesthesiologist physical status classification; CI, confidence interval; eGFR, estimated glomerular filtration rate; mBP, mean blood pressure; *n*, number; OR, odds ratio; RASI, renin–angiotensin system inhibitors; ref, reference.

Factor	Cases (*n*)	Univariate analysis		
		OR	95% CI	*P*-value
Patient characteristics				
Age (years)	133	1.024	0.983–1.067	0.250
Sex	133			
Male		1.000	ref	
Female		0.465	0.148–1.464	0.189
Height	133	1.017	0.974–1.061	0.441
Weight	133	1.018	0.989–1.047	0.220
Body mass index	133	1.071	0.968–1.184	0.180
ASA-PS	133			
1		1.000	ref	
2		0.670	0.080–5.598	0.710
3		0.508	0.039–6.554	0.601
Hemoglobin	133	0.899	0.720–1.124	0.347
Hematocrit	133	0.969	0.893–1.052	0.448
eGFR	130	0.968	0.946–0.991	0.006
≥ 60 mL/min/1.73 m^2^		1.000	ref	
45–60 mL/min/1.73 m^2^		3.131	1.256–7.806	0.015
< 45 mL/min/1.73 m^2^		4.131	1.463–11.664	0.008
Comorbidity	133			
Hypertension		1.064	0.496–2.281	0.872
Heart disease		1.105	0.305–3.994	0.879
Diabetes mellitus		1.511	0.585–3.900	0.391
End stage of renal disease		3.176	0.242–41.632	0.376
Medication	133			
RASI		1.129	0.522–2.444	0.756
Calcium channel blocker		0.964	0.454–2.051	0.925
α-blocker		1.532	0.503–4.662	0.450
β-blocker		0.863	0.220–3.377	0.831
Diuretic		1.239	0.286–5.363	0.773
Perioperative data				
Anesthesia method	133			
General		1.000	ref	
Spinal		1.808	0.689–4.744	0.227
Operation time	133	0.998	0.983–1.013	0.801
Anesthesia time	133	0.997	0.983–1.010	0.625
Preoperative infusion volume	133	1.001	0.999–1.004	0.284
Intraoperative infusion volume	133	1.001	1.000–1.002	0.025
Intraoperative vasopressor	133			
Ephedrine		1.019	0.974–1.065	0.410
Phenylephrine		1.261	0.609–2.610	0.529
Dopamine		4.623	0.407–52.512	0.214
Dobutamine		2.897	0.221–38.051	0.415
Noradrenaline		3.642	0.819–16.186	0.089
Analgesic drug	133			
Fentanyl		0.596	0.235–1.511	0.237
Pentazocine		0.809	0.321–2.040	0.650
Blood pressure	133			
mBP before administration of 5-ALA		0.936	0.899–0.973	0.002
mBP before administration of 5-ALA < 95 mmHg		2.815	1.287–6.158	0.010
mBP upon entering the operating room		0.938	0.906–0.971	< 0.001
mBP upon entering the operating room < 95 mmHg		3.442	1.580–7.497	0.002

**Table 3:** Multivariate analysis of predictive factors of postoperative hypotension Multivariate analyses were performed using mixed models. CI, confidence interval; eGFR, estimated glomerular filtration rate; mBP, mean blood pressure; OR, odds ratio; ref, reference.

Factor	Multivariate analysis		
	OR	95% CI	*P*-value
eGFR			
≥ 60 mL/min/1.73 m^2^	1.000	ref	
45–60 mL/min/1.73 m^2^	3.026	1.140–8.003	0.027
< 45 mL/min/1.73 m^2^	4.851	1.550–15.177	0.007
Blood pressure			
mBP before administration of 5-ALA < 95 mmHg	2.367	0.976–5.740	0.056
mBP upon entering the operating room < 95 mmHg	2.443	1.018–5.865	0.046

**Figure 2:**
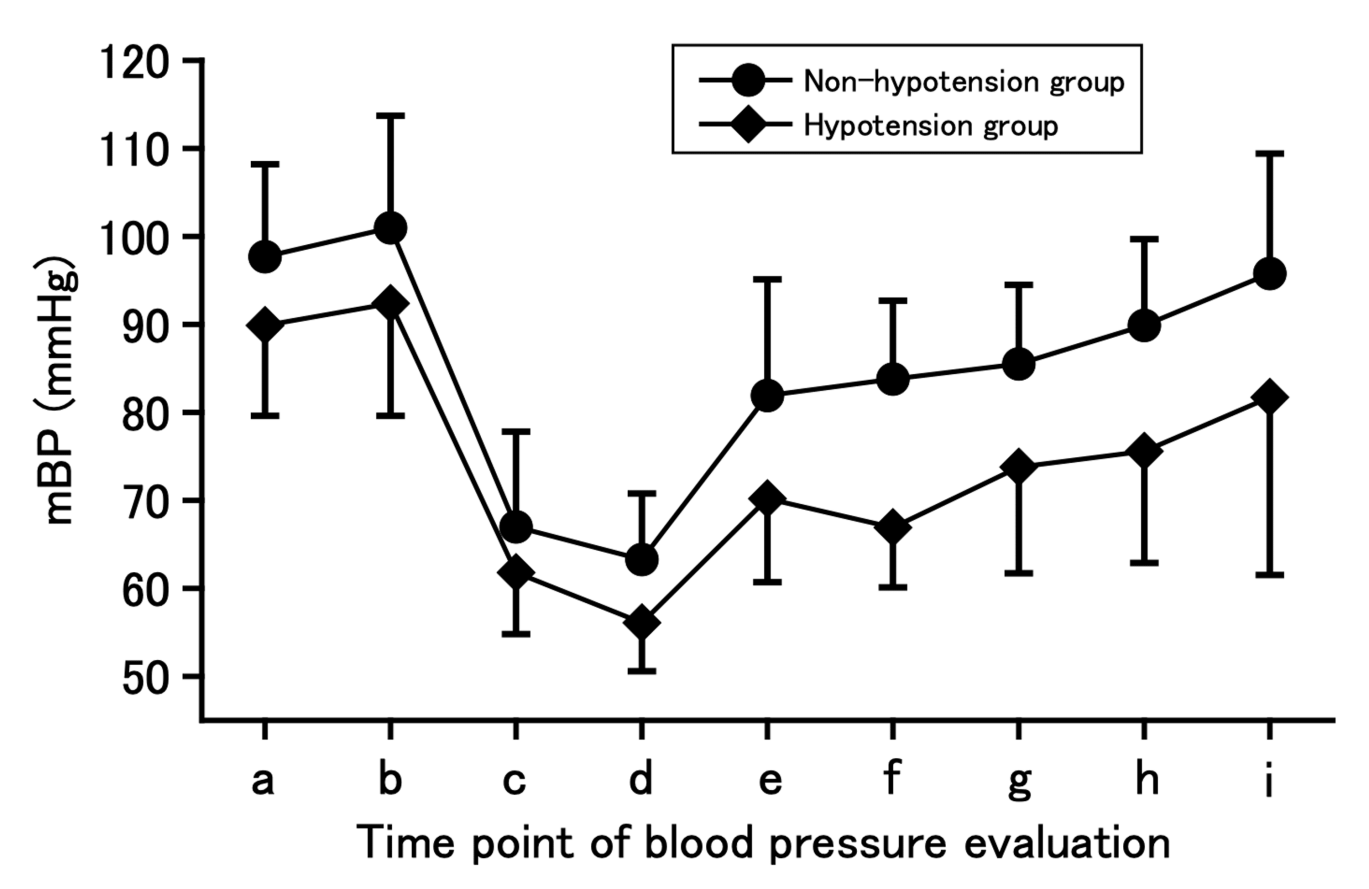
Trends of mean blood pressure in the non-hypotension and hypotension groups of patients who underwent spinal anesthesia. The non-hypotension group: n = 50, and hypotension group: n = 38. In this study using mixed models, patients who underwent two or more TUR-Bt during the study period were also included. For duplicate cases, data from the first case was used. Data are expressed as mean ± standard deviation. mBP, mean blood pressure. Timepoints: a, before administration of 5-aminolevulinic acid; b, upon entering the operation room; c, minimum value before surgery; d, minimum value after starting surgery; e, upon leaving the operation room; f, 1 h after surgery; g, 2 h after surgery; h, 3 h after surgery; and i, 6 h after surgery.

Next, we examined the risk factors for postoperative hypotension in patients undergoing spinal anesthesia (50 patients in the non-hypotension group and 38 patients in the hypotension group). Table 4 shows the patient background characteristics and perioperative information of the two groups. Comparisons between the groups revealed significant differences in weight; eGFR; intraoperative infusion volume; phenylephrine dose; mBP before 5-ALA administration and upon entry into the operating room; minimum mBP before and during surgery; mBP on leaving the operating room and at different time points, 1, 2, 3, and 6 h, after surgery; and use of postoperative vasopressors. The perioperative mBP trends in each group are shown in Figure 2.

**Table 4:** Patient characteristics and perioperative data of spinal anesthesia cases In this study using mixed models, patients who underwent two or more TUR-Bt during the study period were also included. For duplicate cases, data from the first case was used. Fisher’s exact probability test was used for categorical variables, and the Mann–Whitney U test was used for continuous variables. 5-ALA, 5-aminolevulinic acid; ASA-PS, American Society of Anesthesiologist physical status classification; BMI, body mass index; eGFR, estimated glomerular filtration rate; IQR, interquartile range; mBP, mean blood pressure; *n*, number; RASI, renin–angiotensin system inhibitors.

Variables	Non-hypotension group		Hypotension group		*P*-value
	n	Data	n	Data	
Patient characteristics					
Age (years), median (IQR)	50	73.5 (67.0–80.5)	38	74.5 (68.0–80.3)	0.673
Sex	50		38		0.252
Male, n (%)		39 (78.0)		34 (89.5)	
Female, n (%)		11 (22.0)		4 (10.5)	
Height (cm), median (IQR)	50	165.6 (157.0–171.9)	38	163.8 (160.0–167.6)	0.804
Weight (kg), median (IQR)	50	61.4 (52.4–71.9)	38	66.4 (57.9–69.2)	0.045
BMI (kg/m^2^), median (IQR)	50	23.6 (20.6–25.5)	38	24.1 (22.8–26.6)	0.090
ASA-PS	50		38		0.520
1, n (%)		1 (2.0)		4 (10.5)	
2, n (%)		45 (76.0)		27 (71.1)	
3, n (%)		4 (22.0)		7 (18.4)	
Hemoglobin (g/dL), median (IQR)	50	14.2 (13.4–15.4)	38	13.7 (12.3–15.3)	0.225
Hematocrit (%), median (IQR)	50	42.3 (39.9–45.5)	38	40.8 (37.2–45.2)	0.205
eGFR (mL/min/1.73 m^2^), median (IQR)	50	63.0 (54.0–74.5)	38	54.0 (42.3–66.0)	0.011
≥ 60, n (%)		31 (63.3)		13 (36.1)	0.007
45–60, n (%)		12 (24.5)		11 (30.6)	
< 45, n (%)		6 (12.2)		12 (33.3)	
Comorbidity	50		38		
Hypertension, n (%)		31 (62.0)		22 (57.9)	0.826
Heart disease, n (%)		6 (12.0)		3 (7.9)	0.726
Diabetes mellitus, n (%)		7 (14.0)		7 (18.4)	0.770
End stage of renal disease, n (%)		1 (2.0)		2 (5.3)	0.576
Medication	50		38		
RASI, n (%)		20 (40.0)		14 (36.8)	0.827
Calcium channel blocker, n (%)		29 (58.0)		19 (50.0)	0.520
α-blocker, n (%)		4 (8.0)		2 (5.3)	0.695
β-blocker, n (%)		4 (8.0)		4 (10.5)	0.722
Diuretic, n (%)		4 (8.0)		2 (5.3)	0.695
Perioperative data					
Puncture intervertebral	50		38		0.267
< L3/4, n (%)		1 (2.0)		4 (10.5)	
L3/4, n (%)		38 (76.0)		27 (71.1)	
> L3/4, n (%)		11 (22.0)		7 (18.4)	
Baricity of 0.5% bupivacaine	50		38		0.219
Isobaric, n (%)		9 (18.0)		3 (7.9)	
Hyperbaric, n (%)		41 (82.0)		35 (92.1)	
Dose of bupivacaine (mL), median (IQR)	50	2.0 (1.8–2.2)	38	2.0 (1.8–2.1)	0.308
≥ 2.5 ml, n (%)		6 (12.0)		2 (5.3)	0.475
2.5–2.0 ml, n (%)		31 (62.0)		25 (65.8)	
< 2.0 ml, n (%)		13 (26.0)		11 (28.9)	
Preoperative anesthesia level > 10th thoracic vertebra, n (%)	49	25 (51.0)	35	21 (60.0)	0.506
Postoperative anesthesia level > 10th thoracic vertebra, n (%)	43	24 (55.8)	33	23 (69.7)	0.243
Operation time (min), median (IQR)	50	52.0 (34.8–66.8)	38	45.5 (28.8–68.0)	0.433
Anesthesia time (min), median (IQR)	50	72.0 (54.0–87.5)	38	76.5 (49.3–92.0)	0.866
Infusion volume	50		38		
Preoperative infusion volume (mL), median (IQR)		0.0 (0.0–0.0)		0.0 (0.0–162.5)	0.187
Intraoperative infusion volume (mL), median (IQR)		725.0 (517.5–912.5)		875.0 (687.5–1212.5)	0.020
Postoperative infusion volume up to 6 h (mL), median (IQR)		480.0 (480.0–480.0)		480.0 (480.0–492.5)	0.585
Intraoperative vasopressor	50		38		
Ephedrine (mg), median (IQR)		5.0 (0.0–12.0)		8.0 (0.0–16.0)	0.325
Phenylephrine (mg), median (IQR)		0.0 (0.0–0.0)		0.2 (0.0–0.6)	0.002
Dopamine, n (%)		0		2 (5.3)	0.184
Dobutamine, n (%)		0		2 (5.3)	0.184
Norepinephrine, n (%)		1 (2.0)		4 (10.5)	0.161
Sedative drug	50		38		
Propofol, n (%)		46 (92.0)		36 (94.7)	0.695
Excluding propofol, n (%)		14 (28.0)		7 (18.4)	0.325
Analgesic drug	50		38		
Fentanyl, n (%)		1 (2.0)		1 (2.6)	> 0.999
Pentazocine, n (%)		13 (26.0)		9 (23.7)	> 0 .999
Blood pressure					
mBP before administration of 5-ALA, median (IQR)	50	96.3 (91.1–106.2)	38	92.5 (79.9–95.8)	0.001
mBP upon entering the operating room, median (IQR)	50	99.3 (91.6–106.5)	38	91.7 (84.9–101.2)	0.005
Minimum mBP before starting operation, median (IQR)	50	67.0 (60.4–77.7)	38	61.2 (57.3–66.3)	0.001
Minimum mBP after starting operation, median (IQR)	50	62.0 (57.6–66.8)	38	55.3 (52.0–57.7)	< 0.001
mBP upon leaving the operating room, median (IQR)	50	75.0 (70.3–84.7)	38	69.3 (63.5–74.7)	< 0.001
mBP 1 h after surgery, median (IQR)	50	81.2 (76.0–87.8)	38	66.5 (62.6–69.8)	< 0.001
mBP 2 h after surgery, median (IQR)	50	85.6 (78.5–91.3)	38	73.0 (67.9–81.3)	< 0.001
mBP 3 h after surgery, median (IQR)	50	89.3 (83.3–98.0)	38	72.5 (66.4–83.1)	< 0.001
mBP 6 h after surgery, median (IQR)	50	97.0 (87.6–104.4)	37	84.0 (73.0–94.2)	< 0.001
Use of postoperative vasopressors, n (%)	50	0	38	12 (31.6)	< 0.001
Postoperative complications, n (%)	50	3 (6.0)	38	6 (15.8)	0.166
Classification		Nausea and vomiting, 6 cases		Nausea and vomiting, 3 cases; delirium, 1 case; headache, 2 cases	

The results of the univariate and multivariate analyses of patients undergoing spinal anesthesia are shown in Tables 5-6. Univariate analysis revealed significant differences in body weight, BMI, eGFR, intraoperative fluid volume, mBP before 5-ALA administration, and mBP upon entry into the operating room. Multivariate analysis was performed on these factors except for intraoperative fluid volume and revealed significant differences in BMI, eGFR ≤ 45-60 mL/min/1.73 m^2^, eGFR < 45 mL/min/1.73 m^2^, and mBP < 95 mmHg upon entry into the operating room.

**Table 5:** Univariate analysis of predictive factors of postoperative hypotension for spinal anesthesia cases Univariate analyses were performed using mixed models. *Odds ratio could not be calculated because there were no cases of dobutamine use in the non-hypotensive group. 5-ALA, 5-aminolevulinic acid; ASA-PS, American Society of Anesthesiologist physical status classification; CI, confidence interval; eGFR, estimated glomerular filtration rate; mBP, mean blood pressure; *n*, number; n.c., not calculated; OR, odds ratio; RASI, renin–angiotensin system inhibitors; ref, reference.

Factor	Cases (*n*)	Univariate analysis		
		OR	95% CI	*P*-value
Patient characteristics				
Age	105	1.012	0.961–1.066	0.647
Sex	105			
Male		1.000	ref	
Female		0.369	0.103–1.314	0.122
Height	105	1.011	0.965–1.059	0.651
Weight	105	1.043	1.002–1.085	0.038
Body mass index	105	1.196	1.035–1.384	0.016
ASA-PS	105			
1		1.000	ref	
2		0.769	0.090–6.555	0.809
3		0.799	0.057–11.201	0.866
Hemoglobin	105	0.857	0.663–1.107	0.235
Hematocrit	105	0.946	0.859–1.042	0.254
eGFR	102	0.968	0.941–0.995	0.020
≥ 60 mL/min/1.73 m^2^		1.000	ref	
45–60 mL/min/1.73 m^2^		3.072	1.104–8.548	0.032
< 45 mL/min/1.73 m^2^		4.609	1.410–15.067	0.012
Comorbidity	105			
Hypertension		0.982	0.416–2.316	0.966
Heart disease		0.657	0.141–3.063	0.590
Diabetes mellitus		2.178	0.727–6.520	0.162
End stage of renal disease		2.842	0.214–37.718	0.425
Medication	105			
RASI		1.137	0.480–2.694	0.768
Calcium channel blocker		0.653	0.280–1.521	0.320
α-blocker		1.240	0.318–4.840	0.755
β-blocker		1.409	0.300–6.605	0.661
Diuretic		1.409	0.297–6.678	0.663
Perioperative data				
Puncture intervertebral	104			
< L3/4		3.113	0.394–24.618	0.279
L3/4		1.070	0.366–3.130	0.901
> L3/4		1.000	ref	
Baricity of 0.5% bupivacaine	104			
Isobaric		1.000	ref	
Hyperbaric		2.383	0.667–8.511	0.179
Dose of bupivacaine	104	0.815	0.210–3.159	0.765
≥ 2.5 mL		0.660	0.123–3.539	0.624
2.5–2.0 mL		0.881	0.338–2.294	0.793
< 2.0 mL		1.000	ref	
Preoperative anesthesia level > 10th thoracic vertebra	99	1.550	0.647–3.716	0.322
Postoperative anesthesia level > 10th thoracic vertebra	92	1.615	0.625–4.169	0.318
Operation time	105	0.991	0.974–1.008	0.281
Anesthesia time	105	0.994	0.978–1.010	0.439
Preoperative infusion volume	105	1.001	0.998–1.004	0.594
Intraoperative infusion volume	105	1.001	1.000–1.002	0.034
Intraoperative vasopressor	105			
Ephedrine		1.019	0.971–1.070	0.441
Phenylephrine		1.855	0.793–4.338	0.152
Dopamine		3.140	0.233–42.284	0.385
Dobutamine		n.c.*		
Norepinephrine		7.926	0.805–78.009	0.076
Sedative drug during surgery	105			
Propofol		0.853	0.194–3.758	0.832
Excluding propofol		0.722	0.269–1.938	0.515
Analgesic drug during surgery	105			
Fentanyl		1.379	0.069–27.547	0.832
Pentazocine		0.865	0.337–2.217	0.760
Blood pressure	105			
mBP before administration of 5-ALA		0.942	0.903–0.983	0.006
mBP before administration of 5-ALA < 95 mmHg		2.710	1.136–6.468	0.025
mBP upon entering the operating room		0.943	0.908–0.978	0.002
mBP upon entering the operating room < 95 mmHg		3.690	1.535–8.870	0.004

**Table 6:** Multivariate analysis of predictive factors of postoperative hypotension for spinal anesthesia cases Multivariate analyses were performed using mixed models. CI, confidence interval; eGFR, estimated glomerular filtration rate; mBP, mean blood pressure; OR, odds ratio; ref, reference.

Factor	Multivariate analysis		
	OR	95% CI	*P*-value
Body mass index	1.290	1.079–1.542	0.006
eGFR			
≥ 60 mL/min/1.73 m^2^	1.000	ref	
45–60 mL/min/1.73 m^2^	3.757	1.153–12.249	0.029
< 45 mL/min/1.73 m^2^	7.295	1.804–29.501	0.007
Blood pressure			
mBP before administration of 5-ALA < 95 mmHg	2.751	0.931–8.129	0.067
mBP upon entering the operating room < 95 mmHg	3.134	1.061–9.262	0.039

## Discussion

Postoperative hypotension occurred in approximately 40% of patients with TUR-Bt who received oral 5-ALA. Although the patient background predicted an association between intraoperative and postoperative hypotension, intraoperative blood pressure was not included in the univariate and multivariate analyses because, in the present study, we aimed to predict the occurrence of postoperative hypotension from information other than intraoperative blood pressure; particularly, preoperative information and method of anesthesia. Multivariate analysis showed that eGFR ≤ 60 mL/min/1.73 m^2^ and mBP < 95 mmHg upon entry into the operating room were independent risk factors for postoperative hypotension, whereas method of anesthesia was not. These findings imply that poor renal function resulted in increased postoperative hypotension. In the current study, postoperative hypotension was defined in absolute rather than relative value, suggesting that higher blood pressure before anesthesia was less likely to result in postoperative hypotension.

mBP values before anesthesia in the hypotension group were significantly lower than those in the non-hypotension group of patients, irrespective of whether they received general or spinal anesthesia. Additionally, severe hypotension before anesthesia was also observed in a few patients, possibly owing to 5-ALA administration. The intraoperative infusion volume and phenylephrine dose in the hypotension group were significantly higher than those in the non-hypotension group, likely owing to intraoperative hypotension, as evidenced by a median minimum mBP of < 65 mmHg after anesthesia induction. In patients who underwent spinal anesthesia, multivariate analysis showed that BMI, eGFR ≤ 60 mL/min/1.73 m^2^, and mBP < 95 mmHg upon entering the operating room were independent risk factors for postoperative hypotension. However, none of the spinal anesthesia-related factors, such as specific baricity and dose of bupivacaine, lumbar intervertebral space used, or anesthesia level before and after surgery, were found to be risk factors. The risk factors in patients undergoing spinal anesthesia were similar to those of patients who underwent spinal or general anesthesia, and the choice of technique and drug for spinal anesthesia did not appear to be specifically associated with the occurrence of postoperative hypotension.

Several reports have described the risk factors for intraoperative hypotension associated with oral 5-ALA, but none have investigated the incidence of postoperative hypotension. The risk factors for intraoperative hypotension identified in previous reports include general anesthesia, age, female sex, BMI, eGFR, coexisting hypertension, oral calcium channel blocker or RASI treatment, preoperative general condition, and systolic blood pressure < 100 mmHg before anesthesia induction [1,4,13-18,21]. In particular, age > 70-80 years [1,4,12,13,20] and eGFR < 45-60 mL/min/1.73 m^2^ [1,21] have been frequently reported as risk factors. Importantly, BMI, eGFR, and mBP upon entry into the operating room were also associated with postoperative hypotension in the present study. Although higher hematocrit has been associated with greater intraoperative hypotension [22], this was not the case with the occurrence of postoperative hypotension in the present study, wherein the anesthesia method had no effect on the occurrence of postoperative hypotension. However, the effect of 5-ALA on lowering blood pressure continues for more than 9 h [5]; as sympathetic inhibition during spinal anesthesia is expected to continue after surgery, postoperative hypotension should be considered, especially when spinal anesthesia is selected as the method of anesthesia [20]. Although administering spinal anesthesia in patients with obesity, impaired renal function, or mBP < 95 mmHg upon entering the operating room, and especially, a severe decrease in blood pressure after 5-ALA administration, clinicians should be prepared for hypotension to occur postoperatively. Hypotension induced by 5-ALA may involve the activity of its metabolite, protoporphyrin IX, which induces blood vessel dilation [23,24]. Nonetheless, the mechanisms underlying 5-ALA-induced hypotension remain to be further clarified. Therefore, measures to prevent hypotension should be taken with reference to risk factors for perioperative hypotension.

Miyakawa et al. reported risk factors for severe hypotension in TUR-Bt with 5-ALA [21]. They defined severe hypotension cases as patients who required continuous norepinephrine administration. Severe hypotension cases occurred in eight of 128 patients, four of whom required a vasopressor due to prolonged postoperative hypotension. Risk factors for severe hypotension were concluded to be age ≥ 80 years, BMI ≥ 25 kg/m^2^ and eGFR < 45 mL/min/1.73 m^2^, which were the same as in our results, except for age. Therefore, the dose of 5-ALA should probably be adjusted in patients with a combination of these factors.

This study has some limitations. This was a single-center, retrospective study; a multicenter, prospective study is required to obtain high-quality evidence. A consensus on the definition of postoperative hypotension is lacking, which may limit the generalizability of our findings. Additionally, we did not assess the duration of hypotension, which could have provided further insights into its clinical significance. As this study was based on the assumption that hypotension was induced by oral 5-ALA, we did not include oral 5-ALA as a factor for postoperative hypotension in the statistical analysis. Therefore, patients who were not administered oral 5-ALA were not included. As few patients underwent general anesthesia, it can not be stated with certainty that the method of anesthesia has no influence on the occurrence of postoperative hypotension. Although postoperative hypotension should be monitored because it is associated with noncardiac postoperative myocardial damage and death [11,12], this study included data only up to 6 h postoperatively. In future, it would be worth performing a long-term postoperative follow-up and evaluating the outcomes in the hypotension group. 

## Conclusions

Dependent risk factors for postoperative hypotension in patients undergoing TUR-Bt with 5-ALA under general or spinal anesthesia included eGFR and mBP upon entry to the operating room. In spinal anesthesia cases, dependent risk factors were BMI, eGFR, and mBP upon entry to the operating room, but it did not include any of the spinal anesthesia-related factors.

Our findings demonstrated that, regardless of the anesthesia method, impaired renal function increased postoperative hypotension, and higher blood pressure before anesthesia was less likely to result in postoperative hypotension. In cases with a combination of these risk factors, it is prudent to adjust the dose of 5-ALA or avoid its use in the prevention of postoperative hypotension.
